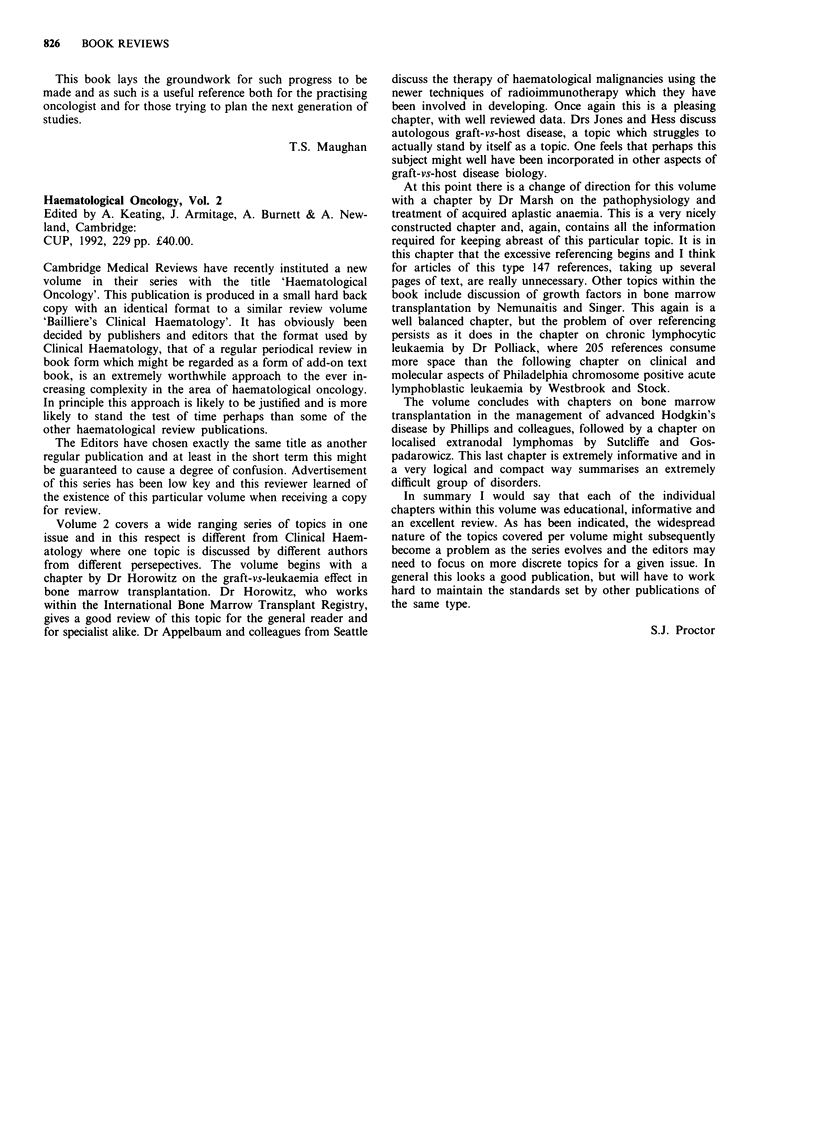# Haematological Oncology Vol. 2

**Published:** 1993-10

**Authors:** S.J. Proctor


**Haematological Oncology, Vol. 2**
Edited by A. Keating, J. Armitage, A. Burnett & A. Newland, Cambridge:
CUP, 1992, 229 pp. £40.00.

Cambridge Medical Reviews have recently instituted a new volume in their series with the title 'Haematological Oncology'. This publication is produced in a small hard back copy with an identical format to a similar review volume 'Bailliere's Clinical Haematology'. It has obviously been decided by publishers and editors that the format used by Clinical Haematology, that of a regular periodical review in book form which might be regarded as a form of add-on text book, is an extremely worthwhile approach to the ever increasing complexity in the area of haematological oncology. In principle this approach is likely to be justified and is more likely to stand the test of time perhaps than some of the other haematological review publications.

The Editors have chosen exactly the same title as another regular publication and at least in the short term this might be guaranteed to cause a degree of confusion. Advertisement of this series has been low key and this reviewer learned of the existence of this particular volume when receiving a copy for review.

Volume 2 covers a wide ranging series of topics in one issue and in this respect is different from Clinical Haematology where one topic is discussed by different authors from different persepectives. The volume begins with a chapter by Dr Horowitz on the graft-*vs*-leukaemia effect in bone marrow transplantation. Dr Horowitz, who works within the International Bone Marrow Transplant Registry, gives a good review of this topic for the general reader and for specialist alike. Dr Appelbaum and colleagues from Seattle discuss the therapy of haematological malignancies using the newer techniques of radioimmunotherapy which they have been involved in developing. Once again this is a pleasing chapter, with well reviewed data. Drs Jones and Hess discuss autologous graft-*vs*-host disease, a topic which struggles to actually stand by itself as a topic. One feels that perhaps this subject might well have been incorporated in other aspects of graft-*vs*-host disease biology.

At this point there is a change of direction for this volume with a chapter by Dr Marsh on the pathophysiology and treatment of acquired aplastic anaemia. This is a very nicely constructed chapter and, again, contains all the information required for keeping abreast of this particular topic. It is in this chapter that the excessive referencing begins and I think for articles of this type 147 references, taking up several pages of text, are really unnecessary. Other topics within the book include discussion of growth factors in bone marrow transplantation by Nemunaitis and Singer. This again is a well balanced chapter, but the problem of over referencing persists as it does in the chapter on chronic lymphocytic leukaemia by Dr Polliack, where 205 references consume more space than the following chapter on clinical and molecular aspects of Philadelphia chromosome positive acute lymphoblastic leukaemia by Westbrook and Stock.

The volume concludes with chapters on bone marrow transplantation in the management of advanced Hodgkin's disease by Phillips and colleagues, followed by a chapter on localised extranodal lymphomas by Sutcliffe and Gospadarowicz. This last chapter is extremely informative and in a very logical and compact way summarises an extremely difficult group of disorders.

In summary I would say that each of the individual chapters within this volume was educational, informative and an excellent review. As has been indicated, the widespread nature of the topics covered per volume might subsequently become a problem as the series evolves and the editors may need to focus on more discrete topics for a given issue. In general this looks a good publication, but will have to work hard to maintain the standards set by other publications of the same type.

S.J. Proctor